# Synthesis of β-cyclodextrin-lysozyme conjugates and their physicochemical and biochemical properties

**DOI:** 10.1007/s10847-017-0706-8

**Published:** 2017-03-08

**Authors:** Tomasz Marek Goszczyński, Maciej Gawłowski, Beata Girek, Konrad Kowalski, Janusz Boratyński, Tomasz Girek

**Affiliations:** 1 Laboratory of Biomedical Chemistry, Department of Experimental Oncology, Hirszfeld Institute of Immunology and Experimental Therapy, PAS, 12 Rudolf Weigl St., 53-114 Wrocław, Poland; 2 Institute of Chemistry, Environmental Protection and Biotechnology, Jan Dlugosz University, Armii Krajowej Ave., 13/15, 42 201 Częstochowa, Poland

**Keywords:** Conjugates, Cyclodextrin, Inclusion complex, Protein, Thermal reaction in solid state

## Abstract

Recently a great interest in the field of protein engineering and the design of innovative drug delivery systems employing specific ligands such as cyclodextrins is observed. The paper reports the solid state, thermal method for protein coupling with β-cyclodextrin and the physicochemical and biological properties of the obtained conjugates. The structure of the obtained conjugates was investigated via liquid chromatography-mass spectrometry, dynamic light scattering and circular dichroism analysis. The presented conjugates were biologically active and covalently bound β-cyclodextrin preserved the ability to form inclusion complexes with the model compound. This report demonstrates the great potential of cyclodextrin as a modifying unit that can be used to modulate the properties of therapeutic proteins, additionally giving such conjugates the possibility to transport many therapeutic substances in the form of inclusion complexes. In addition, the paper presents the potential of protein-cyclodextrin conjugates to construct innovative bioactive molecules for biological and medical applications.

**Electronic supplementary material:**

The online version of this article (doi:10.1007/s10847-017-0706-8) contains supplementary material, which is available to authorized users.

## Introduction

Cyclodextrins (CDs) are cyclic oligosaccharides with significant application in the pharmaceutical, food and cosmetic industries [1, 2]. Naturally occurring CDs are composed of six, seven or eight d-glucopyranoside units in a toroidal structure, possessing a hydrophobic interior and hydrophilic exterior. Accordingly, CDs have an ability to encapsulate and solubilize hydrophobic guest species in water through host–guest complexation [3, 4]. The characteristic properties of the substance, such as solubility, chemical reactivity, or spectral property, are changed after guest compound is encapsulated. Due to these major features, CDs can be utilized in different areas of medicinal chemistry. Recently, these host–guest interactions have been adopted to assemble polymer nanoparticles for drug and gene delivery. Given their biocompatibility, CDs have been used as host units for the synthesis of host–guest delivery carriers. One example of such carriers is diamide linked γ-cyclodextrin (γ-CD) dimers that have been described as molecular-scale delivery capsules for the anticancer agent curcumin [5]. In vivo therapeutic efficacy has been reported for nanoparticles assembled from camptothecin conjugated β-CD polymers [6]. Additionally, cationic β-CD polymer derived nanoparticles have been found to be efficient non-viral delivery vectors for siRNA in humans [7]. Also cyclodextrin-based nanosponges can form complexes with many lipophilic and hydrophilic drugs. They can be used for protecting easily degradable molecules and also could be used to improve the solubility of poorly soluble drugs. For this reason CD-based nanosponges could be used as stable delivery systems and innovative drug carries for therapeutic purposes [8–10].

CDs based rotaxans and polyrotaxanes have been employed as delivery carriers [11, 12]. Furthermore, by affecting the intermolecular interactions of proteins CDs can interfere with their oligomerization and aggregation processes causing a stabilizing effect [13].

Cyclodextrins, due to their possibility to form host–guest complexes with many drugs, can be used to modify the structure of proteins. Supramolecular and covalent CD-protein conjugates has been of great interest in the field of protein engineering. These conjugates are promising for delivery of anticancer drugs [14, 15].

The solid state, thermal reaction between proteins and reducing sugar was first described by Lea [16]. Then, this approach was further developed by Boratyński and Roy [17] as a method for protein glycation and the synthesis of biologically active neoglycoconjugates [18]. Also, experiments on albumin, fibrinogen-methotrexate and lysozyme glycation show that careful choice of temperature and reaction time ensures the retention of the biological activity of the proteins [19, 20]. We have previously reported the usefulness of mono-6-*O*-formyl-β-CD in the modification of proteins in thermal reactions in solid state. In that paper we presented the possibility of creating CD-protein conjugates, and the best conditions for carrying out this reaction have been determined. The obtained CD-BPTI and CD-lysozyme conjugates were characterized by mass spectrometry. MALDI-TOF spectra clearly indicate that in both obtained conjugates, one or two molecules of β-CD to the protein molecule were attached. We proved that thermal reaction in solid state between lysozyme and mono-6-O-formyl-β-CD is temperature and solvent dependent. The use of higher temperature increases the reaction yield but also results in more than one β-CD molecules attached to protein [21].

**Scheme 1:**
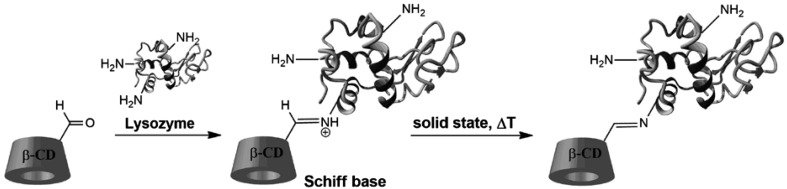
Synthesis of β-CD/lysozyme conjugate

Herein, we report the effect of β-CD conjugation on protein physicochemical and biological properties. As a model protein, we have chosen lysozyme from egg white, molecule with a well-defined structure and biological functions. In our present paper we used DMSO with addition of phosphate buffer (1%) as reaction medium (for high reaction yield) and after freeze drying, temperature 100 °C for 10 min (to obtain predominantly one β-CD molecule per lysozyme). The proposed procedure give a new perspectives for the use this method in the synthesis of biologically active conjugates. The obtained conjugates could be promising in drug delivery systems especially for hydrophobic drugs with low solubility (Scheme 1).

## Experimental

### Reagents and solvents

Crystalline β-CD, lysozyme from chicken egg white, imidazole, p-toluenesulfonyl chloride, collidine (2,4,6-trimethylpyridine), dimethylsulfoxide (DMSO) were obtained from Sigma–Aldrich, St. Louis, USA. Inorganic salts and other solvents were kindly provided by POCH, Gliwice, Poland. Dimethylsulfoxide (DMSO) and collidine were distilled under a vacuum and stored under a 4 Å molecular sieve. Other solvents were used without initial purification. High purity water was generated with a Direct-Q^®^ apparatus (Millipore, Billerica, USA). Quartz cells were used for UV and circular dichroism measurements (Hellma Analytics, Mullheim, Germany).

### Synthesis of mono-6-O-formyl-β-CD

One gram (0.78 mmol) of mono-6-tosyl-β-cyclodextrin, recrystallized twice from hot water, was dissolved in DMSO (10 mL). Collidine (1 mL, 7.6 mmol) was added and the solution was heated at 135 °C for 1.5 h. The resulting slightly brown solution was added to acetone (100 mL) and the white precipitate was collected on a Büchner funnel in a vacuum. The solid was resuspended in acetone (100 mL) and collected in a vacuum. This step was repeated twice. Finally, the white product was dried under a vacuum for 24 h. ^1^H NMR (600 MHz, DMSO-d_6_) δ 9.69 (s, 1 H, CHO), 5.80–5.63 (m, 14 H, OH-2, OH-3), 4.93 (t, J = 4.9 Hz 1 H, H1), 4.82 (d, J = 4.8 Hz, 1 H, H1), 4.44 (t, J = 4.4 Hz, 6 H, OH-6), 4.19 (d, J = 4.18 Hz, 1 H, CHCHO), 3.74– 3.50 (m, 27 H, H1,H3, H6a,b), 3.40–3.25 (m, 14 H, H-2, H-4); ^13^C NMR (150 MHz, DMSO-d_6_) δ 198.8, 102.4, 73.5, 72.9, 72.5, 60.4, 56.5; ESI-MS calcd for C_42_H_68_O_35_ 1132.968, found 1133.357.

### Synthesis of L-CD conjugates via thermal treatment in the solid state

Lysozyme from chicken egg white (10 mg, 0.7 µmol) and mono-6-O-formyl-β-CD (10 mg, 8.8 µmol) were dissolved in dry DMSO (2 mL). The lysozyme and conjugate concentration were determined by measurement of the absorbance at λ = 280 nm, using *ε* = 37 750 cm^−1^ M^−1^ [22]. Finally, phosphate buffer (100 mM, pH = 7.2) was added to the reaction mixture to a final concentration of 1% (v/v). Subsequently, the whole mixture was stirred, frozen in liquid nitrogen and freeze-dried (Christ, Alpha 2–4 LSC, Osterode am Harz, Germany) at 0.1 mBar and room temperature. After freeze drying, the reaction mixtures, in powdered form, were placed in hermetically capped glass tubes under an argon atmosphere. The samples were heated for 10 min in an oven with forced air circulation (Elkon KC-100/200, Lodz, Poland), equilibrated at 100 ± 0.5 °C, then cooled to room temperature and dissolved in acetate buffer (0.4 mL, 100 mM, containing 400 mM sodium chloride, pH 4.0).

### Purification of the L-CD conjugate

Purification was performed using the Ultimate 3000 HPLC system (Dionex, Sunnyvale, USA) and an HIC column (BioSiute Phenyl, 10 µm, 7.5 × 75 mm). Runs were performed at room temperature using a 1.0 mL/min isocratic flow rate of acetate buffer (100 mM, containing 400 mM sodium chloride, pH 4.0). The fraction containing the L-CD conjugate was concentrated by ultrafiltration (Nanosep™ Omega; Pall corp., Port Washington, USA) using 3 kDa cut-off membranes and was then transferred into phosphate buffer (64 mM, containing 10% w/w of glycerol, pH 7.2).

### NMR measurement

NMR spectra were recorded at 600 MHz frequency with an Avance II Bruker Ultrashield Plus spectrometer and a 5 mm sample tube in DMSO-d6 solution without internal standard. All spectra were obtained at ambient temperature. ^1^H NMR and ^13^C NMR spectra were recorded at 600 and 150 MHz, respectively.

### Liquid chromatography-mass spectrometry (LC-MS)

LC-MS analyses were carried out on a MicrOTOF-Q II hybrid quadrupole time-of-flight (Q-TOF) mass spectrometer (Bruker Daltonics, Bremen, Germany) equipped with an electrospray ionization (ESI) source and coupled with an Ultimate 3000 RS HPLC system (Dionex, Sunnyvale, USA). Samples desalting ware performed using a BioBasic column (C8, 300 Å, 5 µm, 2.1 × 50 mm; Thermo Scientific, Waltham, USA) at room temperature using conditions as follows: linear gradient of solution A (0.1% aqueous formic acid (FA)) and B (CH_3_CN with 0.1% FA) from 5 to 70% of solution B in 20 min, flow rate 0.2 mL/min, injection volume 5µL, and detection via MS. All ESI-MS experiments were performed in positive ion mode and calibrated with the sodium formate (10 mM) in water/isopropanol mixture (50/50 v/v) in quadratic + HPC regression mode. Data were acquired with microTOF control 3.0 and processed for calibration and the charge deconvolution of spectra with DataAnalysis 4.0 software (Bruker Daltonics, Bremen, Germany). Standard deviations were estimated by repeated calculations of the average molecular mass of the protein for all ions (*m*/*z*) observed in the protein envelope.

### Ultraviolet spectroscopy (UV) measurements

All the spectrophotometric measurements were conducted on a Specord^®^ 250 (AnalyticJena, Jena, Germany) spectrophotometer equipped with 1.0 cm quartz cells (Hellma Analytics, Mullheim, Germany) at ambient temperature. The absorption spectra have been resolved in the sum of their Gaussian constituents. The process of the decomposition of the absorption spectra of the crystal violet in the sum of the corresponding Gaussians was carried out using Fityk software [23].

### Dynamic light scattering (DLS) measurement

Hydrodynamic parameters of the lysozyme and L-CD conjugates were characterized using the DLS technique. This technique allows for the measurement of time-dependent fluctuations in the intensity of scattered light due to molecular Brownian motion in solution. The analysis of the intensity fluctuations allows the determination of the diffusion coefficients of particles, which are converted into a size distribution (Stokes–Einstein equation). The sample solution was illuminated by a 633 nm laser, and the light intensity scattered at an angle of 173° was measured. At least six consecutive measurements were carried out for each sample. All samples (concentration ca 0.2 mM) were measured at 25 °C (Peltier temperature controller cell holder) in phosphate buffer (64 mM, containing 10% w/w of glycerol, pH 7.2) using a Zetasizer Nano ZS (Malvern Instruments, Worcestershire, U.K.) in a 12-µL quartz cuvette. DLS data were analyzed using DTS 6.10 software (Malvern Instruments, Worcestershire, U.K.). The intensity particle size distributions were obtained using the General Purpose algorithm included in the DTS software. The following parameters were used: protein refractive index (1.450), and solvent viscosity (1.212 × 10^− 4^ Pa × s).

The effect of temperature on the hydrodynamic parameters (determination of melting points - T_M_) of L-CD conjugates was estimated via DLS at 1 °C intervals from 25 to 80 °C and a 3 min equilibrium time at each measurement temperature. The melting point was defined as the temperature at which the hydrodynamic diameter started to increase exponentially with temperature.

### Circular dichroism (CD) measurement

Circular dichroism spectra were recorded at 25 °C on a Jasco 815 spectropolarimeter equipped with a Peltier temperature controller cell holder. Three spectra (recorded with a data pitch of 0.1 nm, a band width of 1 nm, with a detector response time of 4 s at 50 nm/min) were averaged for each sample. Quartz cells with a 1 mm light path length were used for the measurements. Samples were prepared in phosphate buffer (64 mM, containig 10% w/w of glycerol, pH 7.2). Lysozyme and L-CD conjugates concentrations were in the range 6–8 μM for far UV measurements and 60–80 μM for near UV measurements. The mean residue ellipticity ({*θ*}_MRW_) was calculated using the formula: {*θ*}_MRW_ = (*θ*_obsd_MRW)/(10*l*c) where *θ*_obsd_ is the observed ellipticity in degrees, MRW is the mean residue molecular weight (111.8 for lysozyme), *l* is the path length in centimeters, and *c* is the protein concentration in grams per milliliter.

### Biological activity assay

Enzymatic activity of lysozyme and L-CD conjugates was determined using *Micrococcus lysodeikticus* (Sigma–Aldrich, St. Louis, USA) according to the standard procedure [24]. Lytic activity was measured at λ = 450 nm for 5 min (25 °C) in a total volume 2.6 mL of phosphate buffer (66 mM, pH 6.20) containing 0.2 mg/mL of suspended bacteria.

## Results and discussion

### Synthesis of mono-6-O-formyl-β-CD

Mono 6-*O*-formyl-β-CD was obtained by a simple two-step procedure. In the first step a mono 6-*O*-tosyl-β-CD was synthesized by reaction of β-cyclodextrin with 1-(p-toluenesulfonyl) imidazole [25]. In the second step, mono 6-*O*-tosyl-β-CD was oxidized with DMSO as a weak base, afford the monoaldehyde [26]. The obtained mono-6-*O*-formyl-β-CD was characterized by ^1^H and ^13^C NMR (Supplementary material Figures S4 and S5). ^1^H NMR spectra show relevant peaks at 9.7 ppm (CHO), 4.2 ppm (CHCHO) and typical peaks corresponding to β-CD. Within the carbon NMR spectra we were also able to observe an aldehyde peak at 198 ppm. These date were in good agreement with NMR data published previously [21, 27].

### Thermal treatment in solid state

The L-CD conjugates were successfully obtained using thermal reactions in solid state. The physicochemical properties of the obtained conjugates were studied using mass spectrometry (MS), DLS and circular dichroism techniques. It is of interest that no reaction occurs between lysozyme and mono-6-*O*-formyl-β-CD in solution, i.e. in water/DMSO mixture without reducing agent (e.g. NaCNBH_4_), but after freeze drying of the reaction mixture, in solid state and under elevated temperature, it proceeds yielding L-CD conjugates. The solubility of the obtained conjugates in water was comparable with unmodified lysozyme. Thermal treated lysozyme (lysozyme (t): dissolved in water/DMSO mixture, freeze dried, and heated at 100 °C for 10 min) without an addition of the mono-6-*O*-formyl-β-CD shows no essential differences in physicochemical parameters and biological activity as compared to the untreated, control lysozyme (lysozyme(c)).

### LC–ESI–MS analysis

Mass spectrometry is a useful tool to study covalent modification of proteins [28, 29]. Moreover, it has previously been found that the net charge of a protein molecule is sensitive to the protein conformation [30]. Based on the MS data it can be concluded that reaction conditions (elevated temperature in solid state) do not cause any covalent modification or significant change in spatial structure of the lysozyme. Deconvolution of *m*/*z* mass spectra estimated the average molecular mass differences between lysozymes and L-CD conjugates to be 1114.34 and 2228.69 Da, which is equivalent to the mass of a one or two covalently bound β-CD moieties (Table 1).

**Table 1:** Molecular mass differences between lysozyme(c), lysozyme(t) and L-CD conjugates (ESI-MS)

Protein/conjugate	Mprotein/conjugate (Da) calcd/found	ΔMprotein versus conjugate (Da) calcd/found
Lysozyme(c)	14304.98/14305.24	–
Lysozyme(t)	14304.98/14305.67	–
L-CD conjugates	15419.32/15419.48 16533.67/16534.77	1114.34/1114.23 2228.69/2229.52

### Dynamic light scattering analysis

**Fig. 1:**
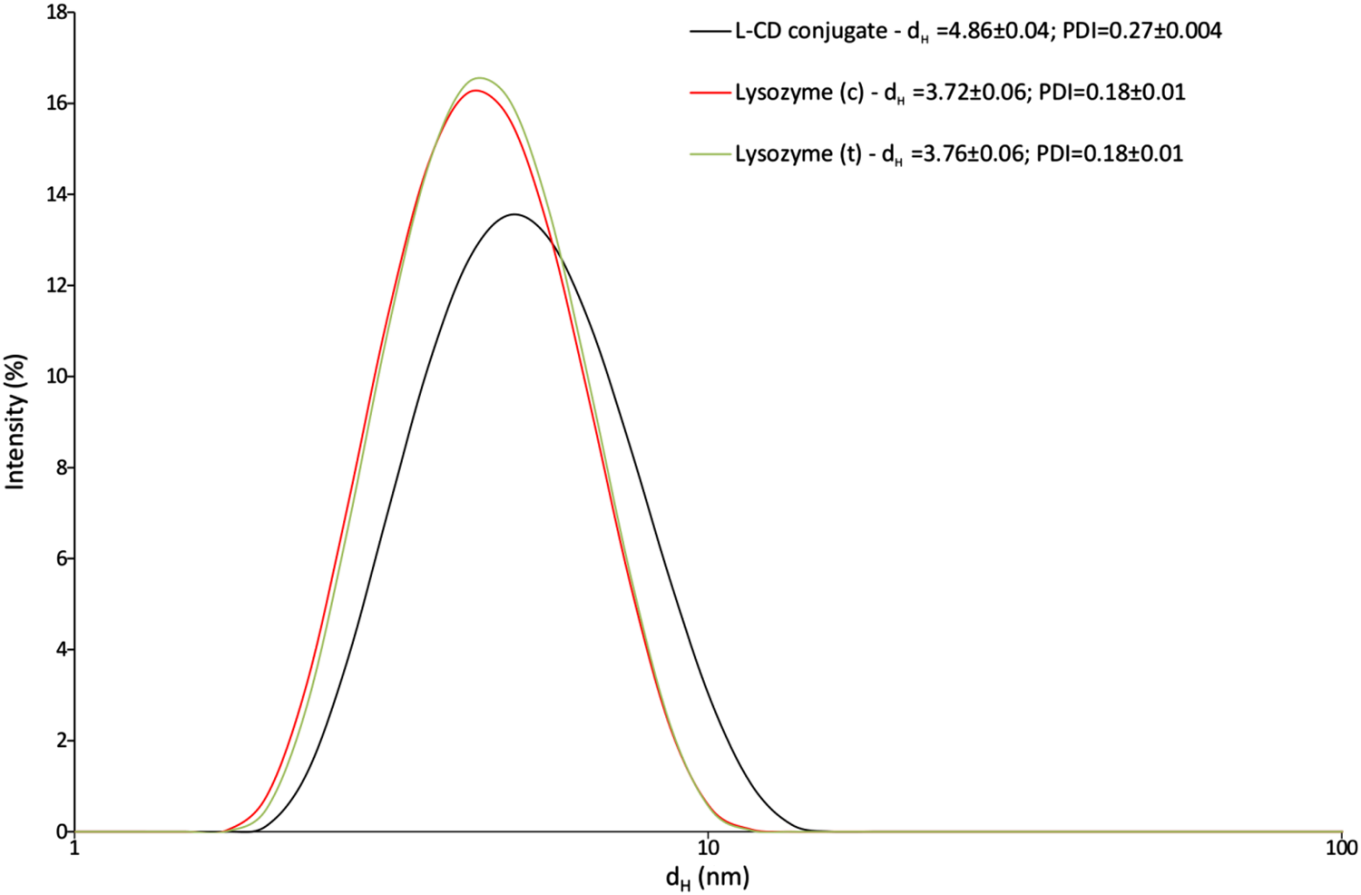
Characterization of lysozyme and L-CD conjugates using the dynamic light scattering technique. Size distributions are shown according to intensity. d_H_—hydrodynamic diameter (nm). Samples (c = 0.2 mM) were prepared in phosphate buffer (64 mM, containing 10% w/w glycerol, pH 7.2)

**Fig. 2:**
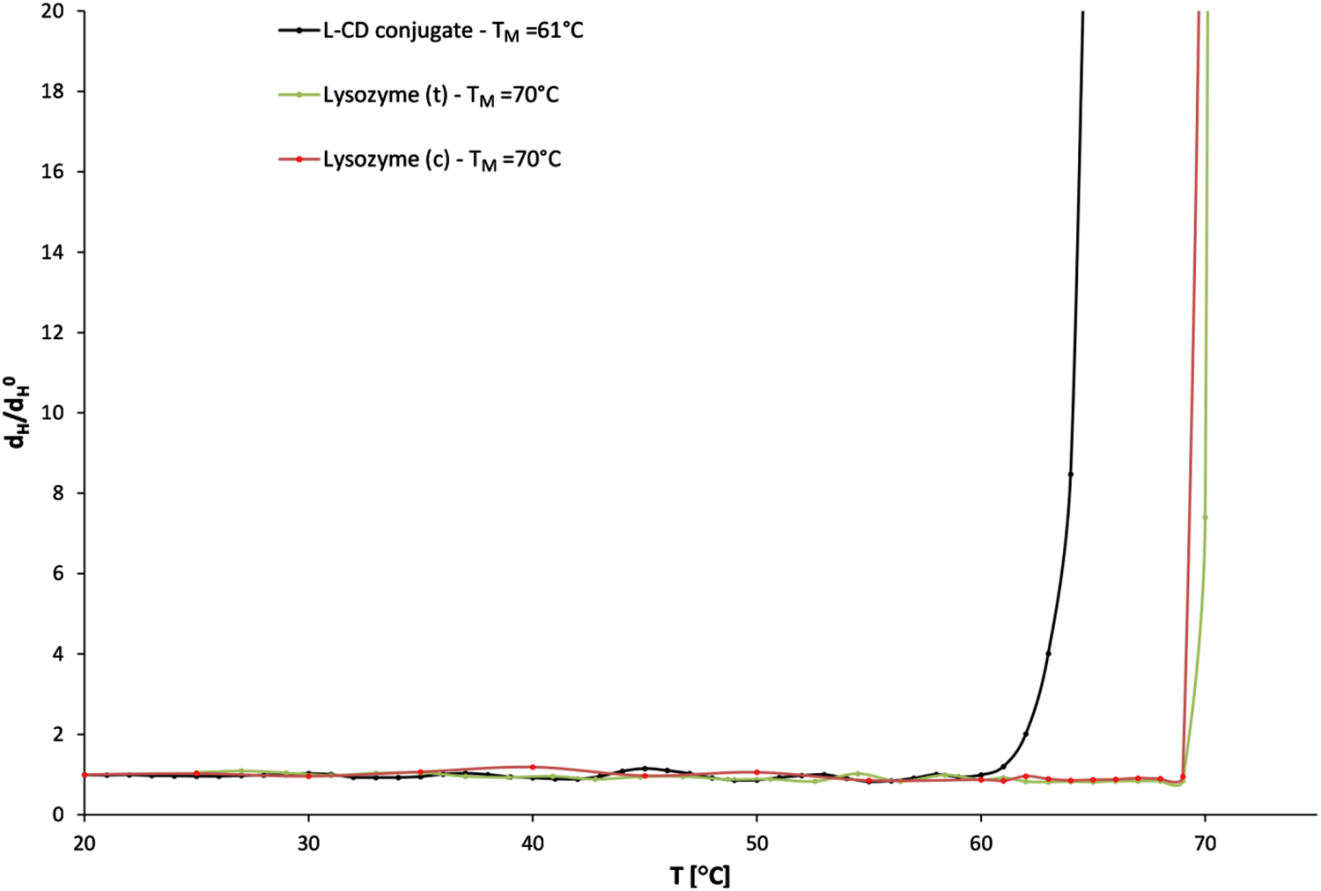
Relative hydrodynamic diameter *d*_H_/*d*_H_^0^ where *d*_H_^0^ is the hydrodynamic diameter of lysozyme and L-CD conjugates at 20 °C determined by the DLS method as a function of temperature. Samples (c = 0.2 mM) were prepared in phosphate buffer (64 mM, containing 10% w/w glycerol, pH 7.2)

The hydrodynamic parameters obtained from DLS measurement are useful for predicting the stability of proteins and their functions [31]. The hydrodynamic diameter and polydispersity of L-CD conjugates are slightly higher than those for unmodified protein (Fig. 1). The changes in hydrodynamic size with temperature are depicted in Fig. 2. The L-CD conjugates exhibit lower thermal stability than lysozyme (c). The melting temperature (T_M_) of lysozyme (c), lysozyme (t) and L-CD conjugates were determined by dynamic light scattering as 70, 70, and 61 °C, respectively.

### Circular dichroism analysis

**Fig. 3:**
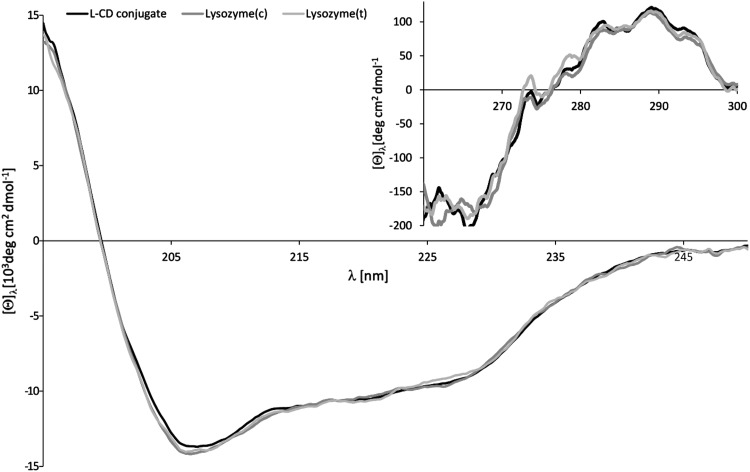
Far and near (inset) UV circular dichroism spectra of lysozyme and L-CD conjugates. Samples were prepared in phosphate buffer (64 mM, containing 10% w/w of glycerol, pH 7.2). Lysozyme and L-CD conjugates concentrations were in the range 6–8 μM and 60–80 μM for measurements in far and near ultraviolet, respectively

The effect of both lysozyme modification with mono-6-*O*-formyl-β-CD and conjugates synthesis conditions (100 °C for 10 min) on the conformational properties of lysozyme, as shown by a comparison of circular dichroism spectra (Fig. 3), seems to be negligible. Based on the near UV circular dichroism data (inset to Fig. 3) it can be concluded that no essential conformational changes were observed for L-CD conjugates, indicating that the cyclodextrin ring did not significantly alter the tertiary structure of lysozyme. In addition circular dichroism measurements proved that thermal reaction conditions do not affect the spatial structure of lysozyme.

### Biological activity of lysozyme and L-CD conjugates

**Fig. 4:**
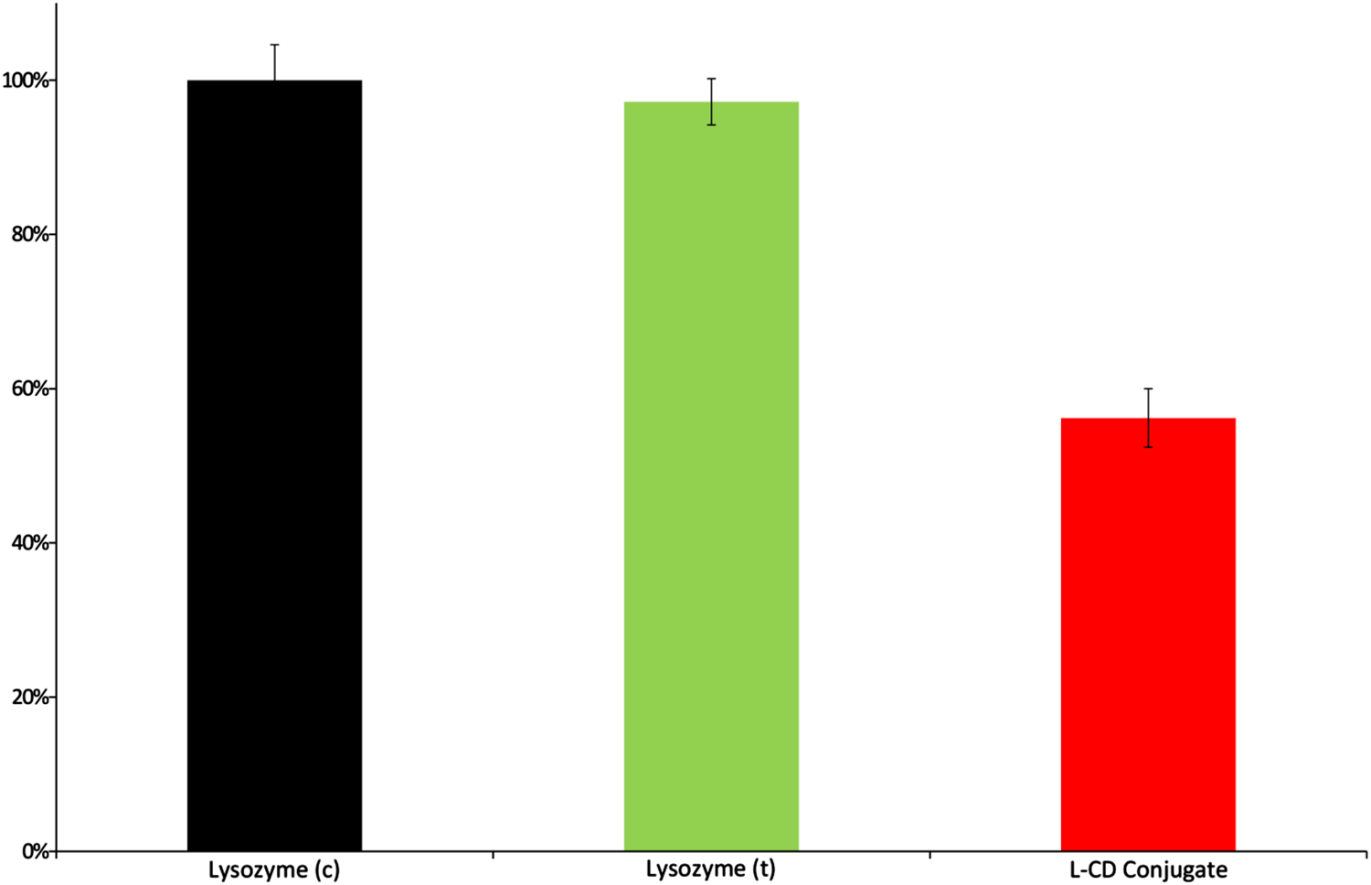
Biological activity of control lysozyme (lysozyme (c)), thermal treated lysozyme (lysozyme (t)) and L-CD conjugates. Enzymatic activity was determined using. *Micrococcus lysodeikticus* (Sigma–Aldrich, ATCC No. 5698, LOT No.:111M8601V) according to the standard procedure [24]

Enzymatic activity analysis of L-CD conjugates shows a decreased ability to hydrolyze bacterial cell walls: 56.2 ± 3.8% as compared to lysozyme(c) (Fig. 4). Lysozyme(t) shows no essential differences in biological activity (97.2 ± 3.0%) as compared to lysozyme(c). These data suggest that reaction conditions (dissolving in water/DMSO mixture, freeze drying, and heating at 100 °C for 10 min) do not affect the biological properties of the lysozyme (measurement variation 3.0%).

The ability to form inclusion complexes between the L-CD conjugates and a model compound (crystal violet) ware investigated via UV–Vis spectrophotometry. Crystal violet (CV), a tris(p-(dimethylamino)phenyl)-methyl ion, is one of the triphenylmethane dyes for which there are many studies concerning molecular structures and complexation processes by β-CD [32]. The visible absorption spectrum of CV in solution appears to be composed of two bands, and their origin was interpreted based on the existence of two isomers or two ground states [33]. CV can form an inclusion complex through the introduction of an aromatic ring in the cavity of the cyclodextrin (absorption signal around λ_1_ = 559 nm and λ_2_ = 596 nm). In this study, we applied visible absorption spectroscopy to investigate the complexes formed by the crystal violet and the β-CD, lysozyme and L-CD conjugates. The absorption spectra have been resolved as the sum of their Gaussian constituents. The process of the decomposition of the absorption spectra of the crystal violet in the sum of the corresponding Gaussians was carried out using Fityk software [23]. The addition of β-CD or L-CD conjugates causes a change in the spectral behavior of CV suggesting that L-CD conjugates can form inclusion complexes with CV. The efficiency of this process is not the same as that of the free cyclodextrin. The reason may be the steric hindrance resulting from the close location of the β-CD ring with a specific region of the protein and hinders CV complexation (Table 2).

**Table 2:** Changes in the spectral behavior of CV (λ_1_ = 559 nm and λ_2_ = 596 nm) under the addition of lysozyme, β-cyclodextrin and L-CD conjugates

Mixture, concentration (µM)	λ_1_ (nm)	λ_1_ (nm)
CV (10 µM)	596.5	559.5
CV (10 µM) + Lysozyme (140 µM)	596.7	559.9
CV (10 µM) + β-CD (140 µM)	598.9	561.4
CV (10 µM) + L-β-CD conjugate (140 µM)	597.2	560.8

The absorption spectra have been resolved in the sum of their Gaussian constituents. Samples were prepared in phosphate buffer (64 mM, containing 10% w/w glycerol, pH 7.2)

In the present study, we demonstrated that lysozyme-cyclodextrin conjugates can be obtained using an innovative method based on thermal treatment in the solid state, without significant secondary or tertiary structure changes of the protein. The obtained conjugates were biologically active (enzymatic activity of lysozyme) and tethered β-CD preserved the ability to form inclusion complexes with the model compound. Additionally, it was reported that mono-6-*O*-formyl-β-CD is a suitable substrate for such reactions. The presented approach also proved a comparable effect on the enzymatic activity of the lysozyme with other methods used for the preparation of lysozyme conjugates with different compounds [34]. Tethering of the β-CD moieties to the lysozyme does not significantly alter its secondary or tertiary structures as indicated by circular dichroism measurements, but does affect its hydrodynamic parameters and thermal stability. Additionally, this paper demonstrates that such conjugates have the possibility to transport various therapeutic substances in the form of inclusion complexes. These factors show the potential of protein-cyclodextrin conjugates for use in biological and medical applications.

## Conclusion

This paper presents the usefulness of solid state, thermal method for the synthesis of biologically active protein-β-CD conjugates. The obtained conjugate system fits in ongoing research concerning development of new drug delivery systems. The systems can extend a new potentiality of chemotherapy, administration of antybiotics or treatment of rheumatological diseases.

## Electronic supplementary material

Below is the link to the electronic supplementary material.

Supplementary material 1 (DOCX 1023 KB)
