# Supplementary material for: Synthesis of β-cyclodextrin-lysozyme conjugates and their physicochemical and biochemical properties
Source: J Incl Phenom Macrocycl Chem. 2017 Mar 8;87(3):341–8. doi: 10.1007/s10847-017-0706-8 (PMC5350216; doi:10.1007/s10847-017-0706-8)
Supplement: Supplementary file 1 — Supplementary material 1 (DOCX 1023 KB) [file 10847_2017_706_MOESM1_ESM.docx]

**Supporting information**

**
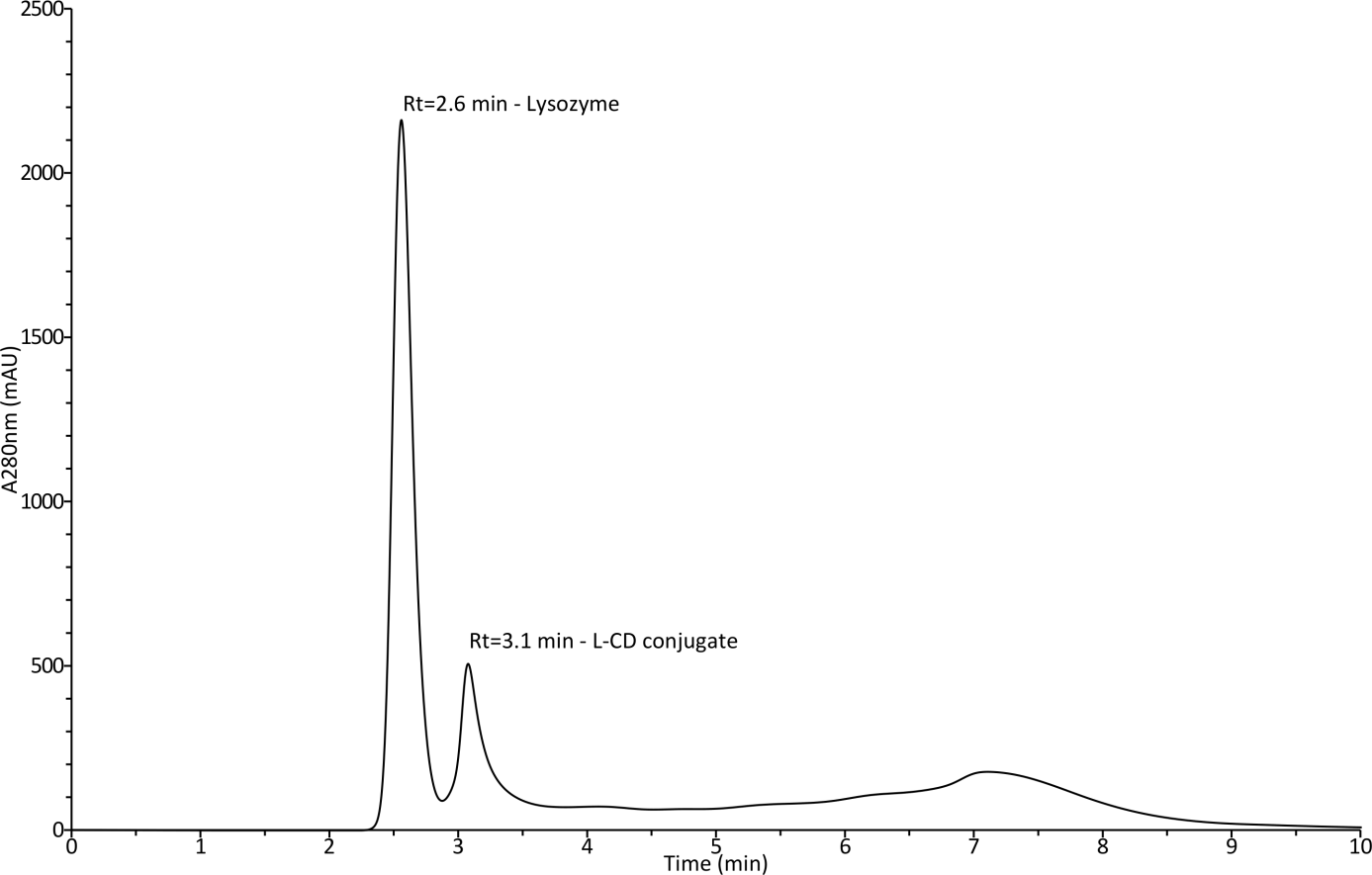
**

**Figure S1.** HPLC profiles of crude L-β-CD product. HPLC analysis was performed on an HIC column (BioSiute Phenyl, 10 µm, 7.5 × 75 mm).

**
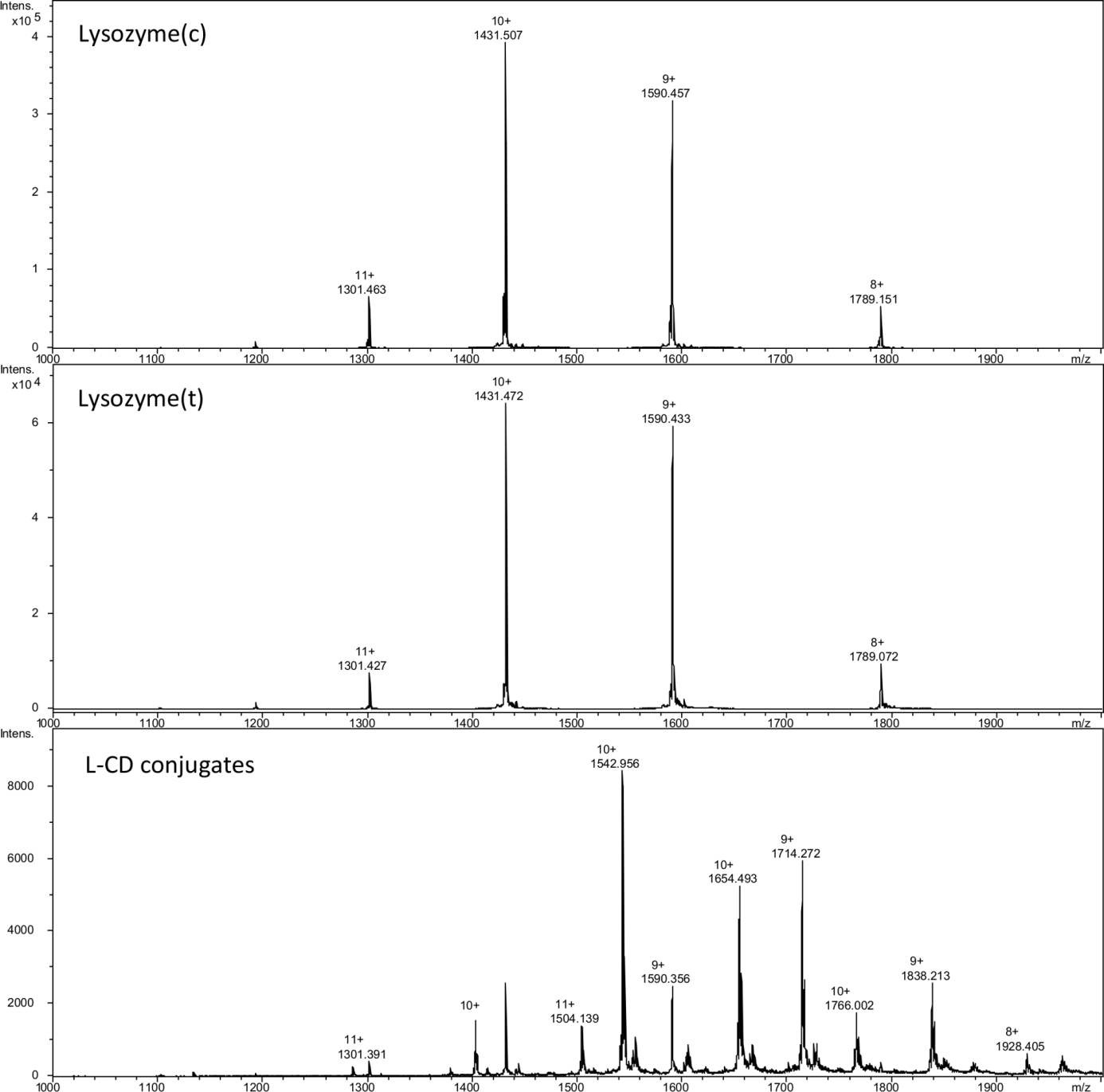
**

**Figure S2.** Comparison of mass spectra (ESI-MS-TOF) of lysozyme(c), lysozyme(t) and L-β-CD conjugates.

**
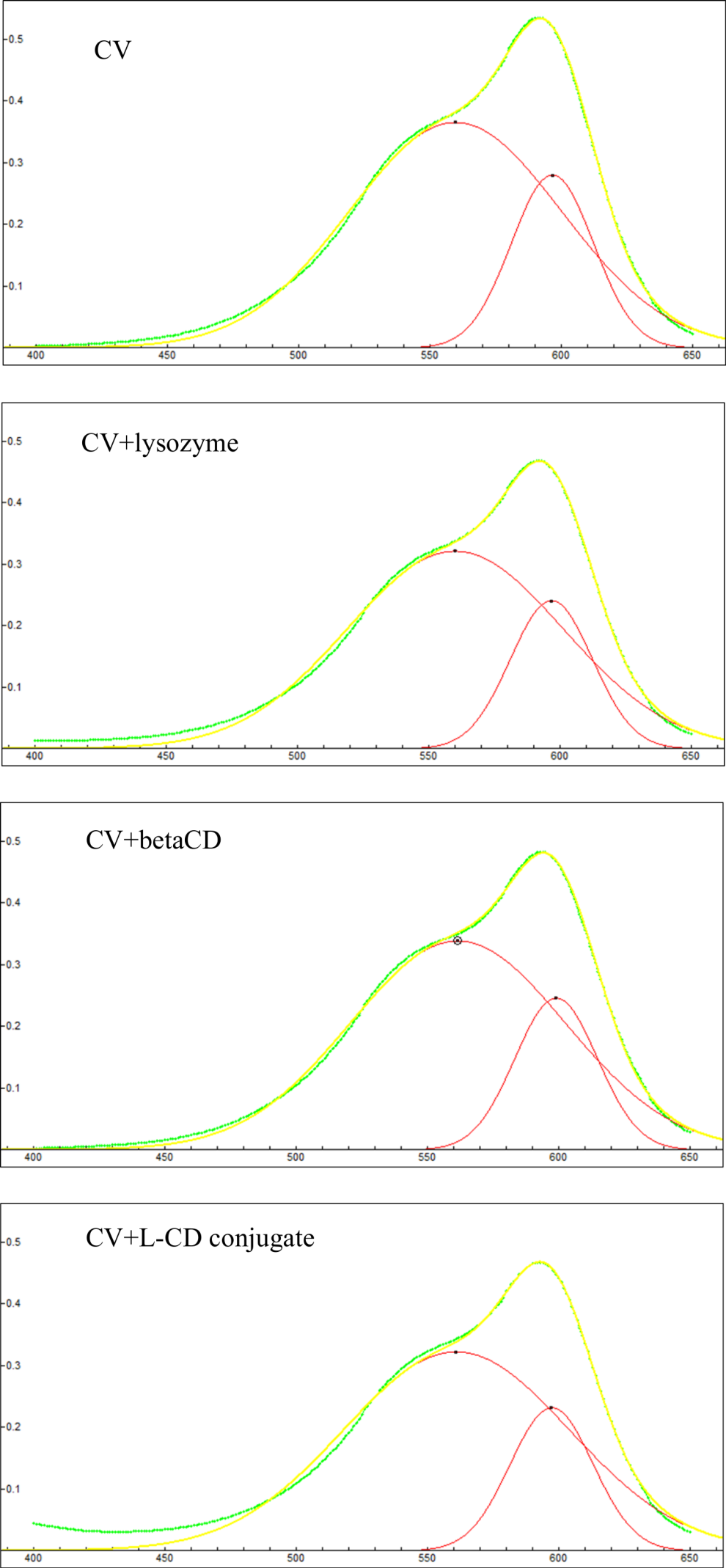
**

**Figure S3.** Decomposition of the absorption spectrum of the crystal violet, in the sum of Gaussians, for the lysozyme, β-CD and L-β-CD conjugates mixtures. Samples were prepared in phosphate buffer (64 mM, containing 10% w/w glycerol, pH 7.2). Green line – measured spectrum, red line – the fitted Gaussians, yellow line – the sum of the Gaussians.

**
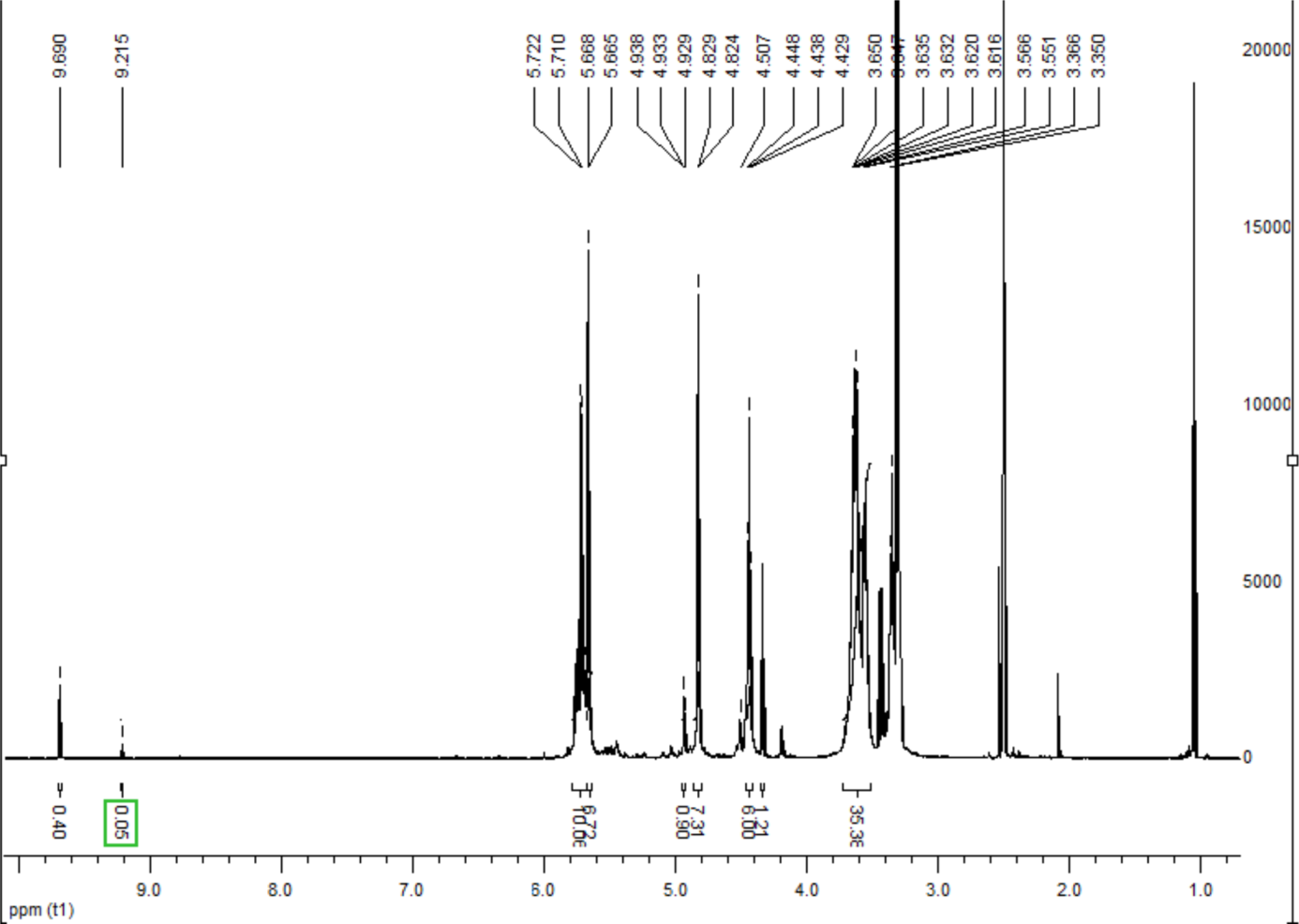
**

**Figure S4.** Proton NMR spectra of mono-6-O-formyl-β-CD.

**
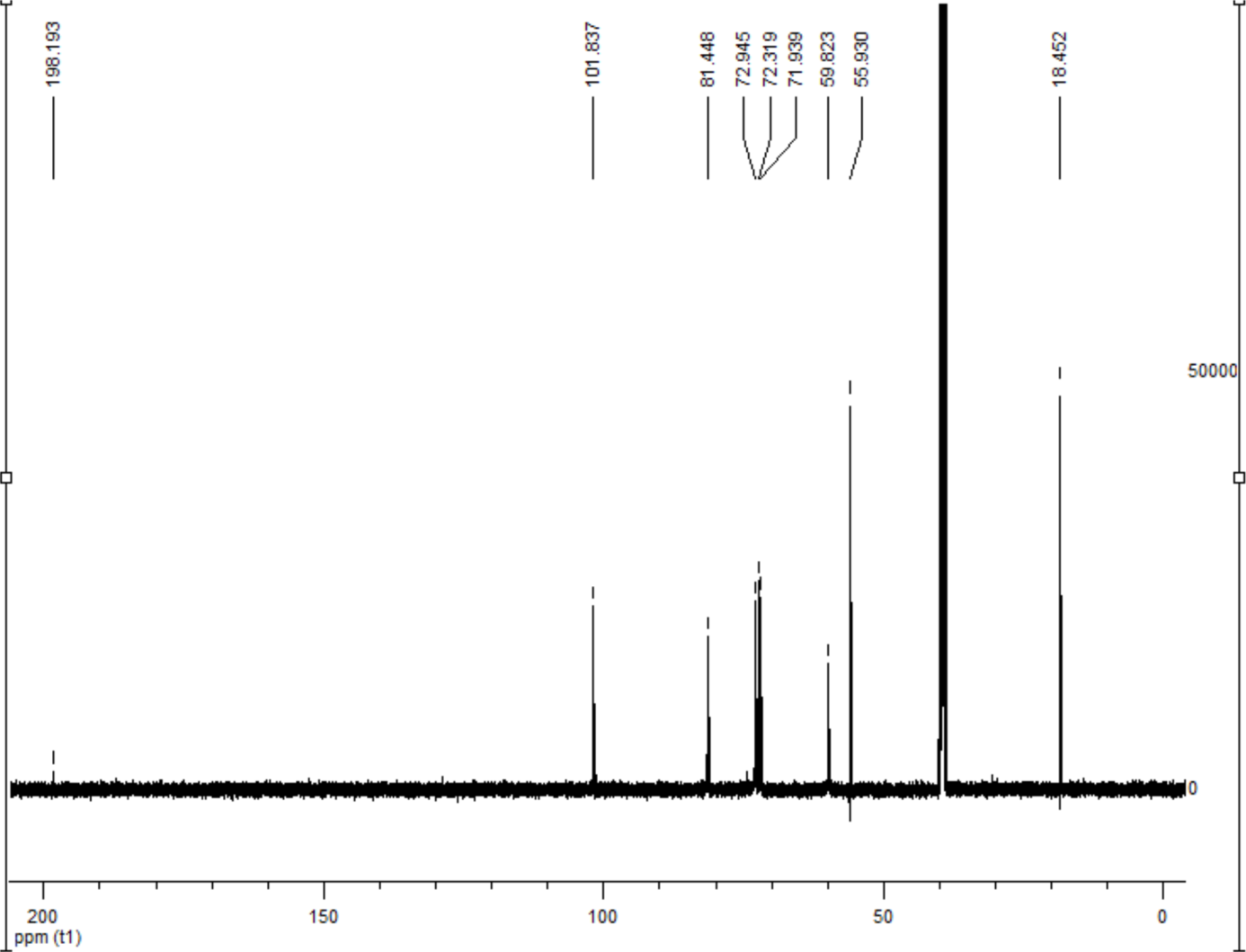
**

**Figure S5.** Carbon NMR spectra of mono-6-O-formyl-β-CD.
